# Measles outbreak investigation in an urban slum of Kaduna Metropolis, Kaduna State, Nigeria, March 2015

**DOI:** 10.11604/pamj.2019.32.150.15764

**Published:** 2019-03-28

**Authors:** Obafemi Joseph Babalola, Ismaila Nda Ibrahim, Ibrahim Usman Kusfa, Saheed Gidado, Patrick Nguku, Adebola Olayinka, Aisha Abubakar

**Affiliations:** 1 Nigeria Field Epidemiology and Laboratory Programme, Abuja, Nigeria; 2 Federal Neuropsychiatry Hospital, Barnawa Kaduna State, Nigeria; 3 Department of Haematology and Blood Transfusion, Ahmadu Bello University, Zaria, Kaduna State, Nigeria; 4 Medical Microbiology and Parasitology Department, Ahmadu Bello University, Zaria, Kaduna State, Nigeria; 5 Department of Community Medicine, Ahmadu Bello University, Zaria, Kaduna State, Nigeria

**Keywords:** Measles, outbreak investigation, routine immunization, urban slum

## Abstract

**Introduction:**

Despite availability of an effective vaccine, the measles epidemic continue to occur in Nigeria. In February 2015, we investigated a suspected measles outbreak in an urban slum in Rigasa, Kaduna State, Nigeria. The study was to confirm the outbreak, determine the risk factors and implement appropriate control measures.

**Methods:**

We identified cases through active search and health record review. We conducted an unmatched case-control (1:1) study involving 75 under-5 cases who were randomly sampled, and 75 neighborhood controls. We interviewed caregivers of these children using structured questionnaire to collect information on sociodemographic characteristics and vaccination status of children. We collected 15 blood samples for measles IgM using Enzyme Linked Immunosorbent Assay. Descriptive, bivariate and logistic regression analyses were performed using Epi-info software. Confidence interval was set at 95%.

**Results:**

We recorded 159 cases with two deaths {case fatality rate = 1.3%}. 50.3% (80) of the cases were male. Of the 15 serum samples, 11(73.3%) were confirmed IgM positive for measles. Compared to the controls, the cases were more likely to have had no or incomplete routine immunization (RI) [adjusted odds ratio (AOR) (95% confidence interval (CI)]: 28.3 (2.1, 392.0), contact with measles cases [AOR (95% CI)]: 7.5 (2.9, 19.7), and having a caregiver younger than 20 years [AOR (95% CI)]: 5.2 (1.2, 22.5). Measles serum IgM was positive in 11 samples.

**Conclusion:**

We identified low RI uptake and contact with measles cases as predictors of measles outbreak in Rigasa, Kaduna State. We recommended strengthening of RI and education of care-givers' on completing RI schedule.

## Introduction

Measles is an acute, highly contagious vaccine preventable viral disease which usually affects younger children. Transmission is primarily person-to-person via aerosolized droplets or by direct contact with the nasal and throat secretions of infected persons [1, 2]. Incubation period is 10-14 days (range, 8-15 days) from exposure to onset of rash, and the individual becomes contagious before the eruption of rashes. In 2014, World Health Organization (WHO) reported 266,701 measles cases globally with 145,700 measles deaths [1]. Being unvaccinated against measles is a risk factor for contracting the disease [3]. Other factors responsible for measles outbreak and transmissions in developing countries are; lack of parental awareness of vaccination importance and compliance with routine immunization schedule, household overcrowding with easy contact with someone with measles, acquired or inherited immunodeficiency states and malnutrition [4-6]. During outbreaks, measles case fatality rate (CFR) in developing countries are normally estimated to be 3-5%, but may reach 10-30% compared with 0.1% reported from industrialized countries [2]. Malnutrition, poor supportive case management and complications like pneumonia, diarrhea, croup and central nervous system involvement are responsible for high measles CFR [7, 8]. Nigeria is second to India among ten countries with large number of unvaccinated children for measles, and has 2.7 million of the 21.5 million children globally that have zero dose for measles containing vaccine (MCV1) in 2013 [9]. Measles is one of the top ten causes of childhood morbidity and mortality with recurrent episodes common in Northern Nigeria at the first quarter of each year [10, 11]. In 2012, 2,553 measles cases were reported in Nigeria, an increase from 390 cases reported in 2006 [10].

In line with regional strategic plan, Nigeria planned to eliminate measles by 2020 by strengthening routine immunization, conduct bi-annual measles immunization campaign for second opportunity, epidemiologic surveillance with laboratory confirmation of cases and improve case management including Vitamin A supplementation. This is meant to improve measles coverage from the present 51% as at 2014 [12] to 95% needed for effective herd immunity. In October 2013, following measles outbreak in 19 States in Northern Nigeria, mass measles vaccination campaign was conducted. The first reported case of a suspected measles in Rigasa community, an urban slum of Kaduna Metropolis occurred on 5^th^ of January 2015 in an unimmunized 10 months old child. The District or Local Government Area (LGA), Disease Surveillance and Notification Officer (DSNO) notified the State Epidemiologist and State DSNO on 10^th^ February, 2015 when the reported cases reached an epidemic threshold. We investigated to confirm this outbreak, determine the risk factors for contracting infection and implement appropriate control measures. This paper describes the epidemiological methods employed in the investigation, summarizes the key findings and highlights the public health actions undertaken.

## Methods

**Study site and study population:** Rigasa is a densely populated urban slum in the south west of Igabi LGA, in Kaduna State, North-West Nigeria. It has an estimated 59,906 households with about 14,156 under-one children. The settlement has three health facilities rendering RI services. The community is noted for poor utilization of RI services and has rejected polio supplemental immunization services in the past. Measles outbreaks have been previously reported from this community. The Last measles supplementary immunization activities (SIA) was conducted from 5^th^ to 9^th^ October, 2013.

**Descriptive epidemiology-quantitative:** in this investigation, a suspected measles case was, any person with generalized maculopapular rash, fever, and at least one of the following; cough, coryza or conjunctivitis or in whom a physician suspected measles, living in Rigasa community, from January 2015 when the index case was reported to March 2015. A confirmed case was, any suspected case with measles IgM positive test or an epidemiological link to a laboratory confirmed case living in the same community at same period. We actively search for cases in the community where measles is locally known as “Bakon dauro”. We interviewed and physically examined some cases at the treatment center to verify diagnosis and ensure that they met the case definition. We developed a line-list to collect information from all suspected cases on their age, sex, residence and time of onset, migration history and immunization status. We analyzed the line-list data to characterize the outbreak in time, place and person, and to develop a plausible hypothesis for measles transmission in the community. We conducted an in-depth interview with health workers rendering routine immunization services to ascertain utilization of RI services by under five-children in the community.

**Case-control study:** we conducted an unmatched case-control study. A suspected case is, any child aged 0-59 months residing in Rigasa with history of fever, rash and at least one of the following: cough, coryza or conjunctivitis or in whom a physician suspected measles; a confirmed case is, positive to measles IgM using enzyme-linked immunosorbent assay (ELISA); and a probable case if there was epidemiological link to a laboratory confirmed measles case. A control was, any child 0-59 months residing in the same community but without the signs and symptoms of measles. We enrolled 75 cases and 75 controls to identify an odds ratio of 3 (for a risk factor on which intervention would have a significant impact), assuming 21.2% prevalence of exposure among control [4], with 95% CI and power of 80%. The sample size was determined using the Statcal function of Epi-Info statistical software. We selected and recruited the cases consecutively from among the patients that presented at the health facility and in the community. The controls were selected from the community; each control was selected from the 3^rd^ homestead to the right of the household of a case. We used structured questionnaires to collect data on demographic characteristics, exposures, vaccination status and associated factors from both cases and controls, and clinical information from the cases only.

**Sample collection and laboratory analysis:** we collected blood samples for 15 suspected cases for measles serum IgM determination using ELISA method at the WHO regional laboratory at Kaduna.

**Data management:** we entered data into Epi-Info statistical software and performed univariate analysis to obtain frequencies and proportions, and bivariate analysis to obtain odds ratios and determine associations, setting p-value of 0.05 as the cut-off for statistical significance. We also performed unconditional logistic regression to adjust for possible confounders and identify the independent factors for contracting measles infection. Factors that were significant at p < 0.05 in the bivariate analysis and biological plausible variable such as age and sex were included in the model. In the final model, only variables that were found to significantly affect the outcome at P < 0.05 were retained. Data management was done using Epi InfoTM software 3.5.3 (CDC Atlanta, USA), and Microsoft Excel. For qualitative study, content analysis was thematically analyzed.

**Ethical consideration:** ethical approval was not obtained as study was conducted as part of an outbreak response. However, permission to conduct the study was granted by the State Primary Health Care Agency, District or LGA Director of Primary Health Care and District head of Rigasa community. Informed consent was obtained from all respondents interviewed. Confidentiality of all the subjects was assured and maintained during and after the study.

## Results

**Figure 1:**
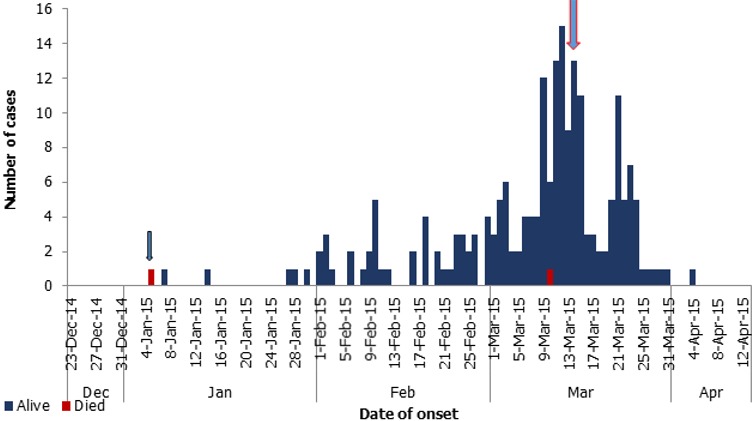
Epidemic curve of measles outbreak in Rigasa community, week 1 to 15, 2015

**Descriptive epidemiology-quantitatively:** a total of 159 cases with two deaths (CFR = 1.3%) were identified. 80 (50.3%) were male. Cumulatively, under-five children accounted for 90% of cases. The mean age of cases was 32 (± 22.5) months but the age group of 0-11 months were severely affected with age specific death rate of 5% (Table 1). The index case for this outbreak was a 10 months old female child that had maculopapular rash on 5^th^ January, 2015. She was unvaccinated for measles and never had any immunization for vaccine preventable diseases according to Expanded Programme on Immunization (EPI) schedule. She had no history of contact with someone with measles and had not travelled out of the community in the last one month. She was managed as an outpatient and died 3 days later. Figure 1 shows the epidemic curve of the outbreak. The epidemic curve has a propagated pattern with four peaks, the highest on 10^th^ March, 2015. The outbreak spanned from 5^th^ January to 4^th^ April, 2015.

**Table 1:** Age distributions of measles cases and case fatality rate (CFR) in Rigasa community, March 2015

Age (Months)	Frequency n (%)	Age specific CFR n (%)
0 - 11	22 (14)	1 (5)
12 - 23	32 (20)	0
24 - 35	42 (26)	1 (2.4)
36 - 47	22 (14)	0
48 - 59	25 (16)	0
More than 59	16 (10)	0
**Total**	**159 (100)**	**2 (1.3)**

**Descriptive epidemiology-qualitative evaluation of RI services:** content analysis of in depth interview of three health workers rendering routine immunization services revealed that caregivers' usually utilized RI services in the first 6-10 weeks of life, no stock out of measles vaccine in last 6 months and most caregivers failed to comply with EPI schedule. Also, all planned RI sessions were held in the past 3 months and there was no vaccine stock out in 6 months prior to the outbreak and the facilities have functioning cold chain system.

**Analytic study:** total population sampled was 150 (cases 75, control 75). The mean age for cases and controls was 33.2 (± 21) and 37.6 (± 29) months. Males were 42 (56%) cases and 37(49%) controls. Among the cases, 15 (20%) was vaccinated for measles compared to 23 (30.7%) of the control. Among the 112 children unvaccinated for measles, attack rate was 54%. Cumulatively, 41(27%) were vaccinated for measles. Only 1 (1.3%) case compared to 12 (16%) of controls had completed the Expanded Programme on Immunization schedule (Table 2). From bivariate analysis, cases were more likely than control, to have had none or incomplete RI [OR (95% CI)]:14.0 (1.8, 111.4); not to have received measles vaccination [OR (95% CI)]:2.0 (0.8, 3.7); to have had close contact with measles cases [OR (95% CI)]:6.0 (2.7, 11.2); and to have caregivers who were younger than 20 years [OR (95% CI)]:2.6 (1, 6.8) (Table 3). From modelling, independent predictors of measles transmission in Rigassa, an urban slum in Kaduna metropolis were none or incomplete routine immunization (RI) [adjusted odds ratio (AOR) (95% confidence interval (CI)<]:28.3 (2.1, 392.0), unvaccinated for measles [AOR (95% CI)]:1.8 (0.8, 3.7), recent contact with measles cases [AOR (95% CI)]:7.5 (2.9, 19.7), and having a caregiver younger than 20 years [AOR (95% CI)]:5.2 (1.2, 22.5) (Table 4).

**Table 2:** Characteristics of measles cases and controls in Rigasa community, March 2015

Characteristics	Case n (%)	Control n (%)	Total n (%)
**Age (Months)**			
0-11	9 (12)	8 (11)	17 (11)
12-59	57(76)	59 (78)	116 (78)
> 59	9 (12)	8 (11)	17 (11)
Sex			
Male	42 (56)	37 (49)	79 (53)
Female	33 (44)	38 (51)	71 (47)
**Measles vaccination status**			
Yes	17 (23)	24 (32)	38 (25)
No	58 (77)	51 (68)	112 (75)
**Complete EPI Schedule**			
No	74 (98.6)	63 (84)	137(91.4)
Yes	1 (1.4)	12 (16)	13 (8.6)
**Recent contact with Measles case**			
Yes	56 (75)	26 (35)	82 (55)
No	19 (25)	49 (65)	68 (45)
**More than 5 children playing together in the compound**			
Yes	34 (45)	16 (21)	50 (33)
No	41 (55)	59 (79)	100 (67)

**Table 3:** Factors that may be responsible for measles outbreak at Rigasa community, Igabi LGA Kaduna State-March 2015

Risk factors of Measles	Case (n=75)	Control (n=75)	Odd’s Ratio	95% CI		P-Value
				Lower	Upper	
**Routine immunization status**						
None or Incomplete	74	63	14	1.8	111.4	<0.01
Complete	1	12				
**Recent Contact with someone with measles**						
Yes	56	26	5.6	2.7	11.2	<0.01
No	19	49				
DPT3						
Unvaccinated	64	44	4	1.9	9	<0.01
Vaccinated	11	31				
**Number of children age <15 Years playing together in the compound**						
More than 5	34	16	3.1	1.5	6.3	<0.01
Less than 5	41	59				
**Malnutrition (MUAC by age and sex)**						
Below 15^th^ percentile	12	5	2.7	0.9	8	0.12
Above 15^th^ percentile	63	70				
**Mother's age**						
Less than or equal to 20 years	16	7	2.6	1	6.8	0.07
More than 20 years	59	68				
**Measles Vaccine**						
Unvaccinated	60	52	1.8	0.8	3.7	0.19
Vaccinated	15	23				
**Mother's education status**						
None or less than 6 years of education	47	48	0.9	0.48	1.83	1
More than 6 years of education	28	27				

**Table 4:** Factors responsible for measles outbreak’s transmission at Rigasa community, Kaduna metropolis, Kaduna State - March 2015

Risk factors	Case	Control	AOR	95% CI		P-Value
				Lower	Upper	
Never/Incomplete EPI Schedule	74	63	14.0	1.8	111.4	<0.01
Recent contact with case	56	26	5.6	2.7	11.2	<0.01
Unvaccinated with DPT-3	64	44	4.0	1.9	9.0	<0.01
More than 5 children in a compd.	34	16	3.1	1.5	6.3	<0.01
Age of caregivers ≤ to 20 years	16	7	2.6	1.0	6.8	0.07
Unvaccinated for measles	58	51	1.6	0.8	3.3	0.27

**Laboratory findings:** eleven (73%) of the 15 serum samples were confirmed IgM positive for measles. Two samples that were negative for measles IgM were also negative of rubella IgM; one was indeterminate, but there was an epidemiological link with a confirmed case of measles. The outcome of the last sample could not be ascertained.

## Discussion

This laboratory confirmed that measles outbreak caused severe morbidity in a densely populated urban slum community in Kaduna metropolis where substandard housing and poor living conditions existed. In addition, the community, like most cosmopolitan settlements in Northern Nigeria, has witnessed poor uptake of RI for all antigens. In this investigation, measles vaccination coverage was 27%; this is lower than the estimated national coverage of 51% [12]. The outbreak of measles in Rigasa spanned from 5^th^ January to 4^th^ April, 2015, weeks after outbreak investigation and response. This prolonged spread of the infection could be due to lack of herd immunity in the community. The case fatality of 1.3% in this study is similar to 1.5% reported in Lagos, a cosmopolitan city in Nigeria, but lower than 3.9% reported in Bayelsa-South-South Nigeria, and 6.9% reported in a rural community in Southwestern Nigeria [8, 11, 13]. The lower CFR reported in this study could be due to early presentation of affected children to health facilities, and improved case management. During the outbreak, the state primary health care agency supplied drugs, including vitamin A, to health facilities that were used in the treatment of cases at no cost. Although measles usually affects under-five children [11], in this study we found under-one to be severely affected with CFR of 5%. In developing countries, malnutrition, lack of supportive care, crowding and poorly managed complications of measles have been implicated as causes of high CFR [8]. Routine immunization is significantly associated with reduction in measles infection among vaccinated individuals. We found that those who had no or incomplete vaccination according to EPI schedule were fourteen times more likely to have measles infection (95% CI. 1.8-111.4). In this community only 8.6% of the children had complete EPI schedule. This was by far less than the national target of at least 87% of RI coverage, and in which no fewer than 90% of the LGAs reach at least 80% of infants with complete scheduled of routine antigens by 2015 [14]. Countries with a single-dose of measles are said to be poor, least developed, report the lowest routine vaccination coverage and experience high measles diseases burden [15]. During the study period, on the EPI schedule, a single dose of measles antigen should be administered to a child at age nine months. Apart from the country's low measles vaccination coverage, primary vaccine failure occurs in 25% of children vaccinated at 9 months, and thus are unable to develop protective humoral antibodies against measles virus [10].

We also found that caregivers who were 20 years or less were more likely to have children with measles. This could be attributed to lack of knowledge on the importance of routine immunization and childcare. Caregivers in this community usually present their children for immunization according to EPI in the first 3 months of life but failed to complete the schedule therefore missing measles vaccination at the ninth month. Reasons put forward by caregivers to why their children missed measles vaccination ranged from being unaware of EPI schedule for measles, to competing priorities, to adverse events following immunization (AEFI). These reasons are similar to those cited in previous literatures [8, 16]. In this outbreak, the epi-curve revealed a propagated epidemic pattern which probably affirms that the disease was transmitted from person to person. Rigasa is a densely populated urban slum community and this allow for the spread of measles in this community. Overcrowding is an important risk factor in the transmission of respiratory infections [17]; and measles being a highly contagious disease, recent contact and overcrowding are risk factors for disease transmissions during measles outbreak [1, 3]. This outbreak response had one major limitation; that is, delay in reactive vaccination in the community after the investigation due to fear of potential postelection violence. This investigation was conducted just before 2015 national election, and the past elections in Nigeria, especially the preceding 2011 election, were marred by postelection violence. However, during the investigation, with support from the traditional leaders and government we were able to implement the following public health actions: educate the community on the importance of measles vaccination, prompt identification of cases in the community and referral to health facilities where drugs supplied by the state government for free treatment of cases were available. A retroactive measles vaccination campaign was carried out in the community after the 2015 national election.

## Conclusion

We confirmed there was a measles outbreak in Rigasa community. Low measles vaccine coverage as a result of poor uptake of RI was responsible for the outbreak of measles infection in the community. This resulted in the accumulation of susceptible children thus lowering the herd immunity against measles infection. This study also suggests that children of younger caregivers were more afflicted with measles infection during this outbreak. The poor housing conditions and overcrowding in this community greatly fueled the outbreak, supporting the evidence that close contact with a measles case is a risk factor for measles transmission. A major public health implication of this study is the need to strengthen RI services. We therefore recommend that the state ministry of health should increase demand creation for RI services through more sensitization and education of caregivers. Additionally, health workers should encourage and motivate caregivers who access RI services to complete the EPI schedule to prevent vaccine preventable diseases. Engaging caregivers who had completely immunized their children according to EPI schedule as a community role model could be a good strategy to motivate other caregivers to access and complete RI services.

### What is known about this topic

- Measles is a highly contagious vaccine preventable viral disease that usually affects younger children;
- Several factors such as low coverage for measles antigens and overcrowding have been noted to be risk factors for measles outbreak.

### What this study adds

- We found children less than one year to be severely affected by measles and having the highest case-fatality;
- In this investigation, we found measles vaccination coverage to be 27% in this Rigasa; this is less than the reported national coverage of 51%;
- This study also reveals that younger caregivers who are 20 years or less compared to older ones, are more likely to have children with measles.

## Competing interests

The authors declare no competing interests.
